# Association between ultra-short-term heart rate variability of time fluctuation and atrial fibrillation: Evidence from MIMIC-IV

**DOI:** 10.1016/j.hroo.2025.03.006

**Published:** 2025-03-14

**Authors:** Xiaodi Tang, Yue Wu, Xiaofei Zhang, Kexin Zhang, Ying Xie, Yangong Chao, Rong He, Ping Zhang

**Affiliations:** 1 Department of Cardiology, Beijing Tsinghua Changgung Hospital, School of Clinical Medicine, Tsinghua Medicine, Tsinghua University, Beijing, China; 2 Department of Cardiology, People’s Hospital of Xiangxi Tujia and Miao Autonomous Prefecture, First Affiliated Hospital of Jishou University, Jishou, China; 3 Department of Critical Care, Beijing Huaxin Hospital First Hospital of Tsinghua University, Beijing, China

**Keywords:** Atrial fibrillation, Ultra-short-term heart rate variability, Autonomic nervous system, Frequency domain metrics, Time domain metrics

## Abstract

**Background:**

Ultra-short-term heart rate variability (usHRV) has been found to be associated with atrial fibrillation (AF); however, research in this area is currently limited.

**Objective:**

This study aimed to investigate the association between usHRV metrics and AF.

**Methods:**

This retrospective cohort study included 48,416 participants from the Medical Information Mart for Intensive Care IV (MIMIC-IV) database. UsHRV time domain and frequency domain metrics were also collected. We examined the connection between usHRV and AF in electrocardiogram samples collected from 08:00 to 18:00, 18:00 to 08:00, and all day to understand the impact of time on the findings. To address the research objectives, we used Cox regression analysis, stratified curve fitting, threshold effect analysis, subgroup analysis, and the assessment of interaction effects.

**Results:**

During an average follow-up of 1.88 years, 3611 (7.5%) participants developed AF. UsHRV time domain metrics were unstable in a day, and the HR was more significant in those ≥55 years of age, with a statistically significant interaction. In contrast, the usHRV frequency metrics are more clinically significant and stable. The hazard ratios for the 08:00 to 18:00 samples were 0.79 (95% confidence interval [CI] 0.74–0.84) for log(ratio of low frequency and high frequency), 0.74 (95% CI 0.67–0.80) for log(low-frequency normalized units), and 2.26 (95% CI 1.80–2.84) for log(high-frequency normalized units), respectively.

**Conclusion:**

The frequency domain metrics of usHRV exhibit strong stability, surpassing those derived from time domain metrics, and offer improved convenience compared with HRV. This makes them particularly notable for their clinical significance.

**Key Findings**

▪ For the association between ultra-short-term heart rate variability (usHRV) and atrial fibrillation (AF) risk, (1) usHRV metrics, particularly frequency domain measures (eg, low-frequency [LF] normalized units, high-frequency [HF] normalized units, and LF/HF ratio), are strongly associated with AF risk; and (2) frequency domain metrics showed greater stability and clinical significance compared with time domain metrics.
▪ For age and usHRV interaction, (1) the association between usHRV time domain metrics and AF risk varied by age, with stronger effects observed in individuals ≥55 years of age; and (2) frequency domain metrics showed consistent protective effects across all age groups.
▪ With regard to clinical implications, (1) usHRV frequency domain metrics, especially LF normalized units and HF normalized units, are robust indicators of AF risk and may be useful for early risk stratification; and (2) the findings support the use of usHRV in large-scale studies and wearable devices for AF prediction.

## Introduction

Heart rate variability (HRV) monitoring serves as a noninvasive method for assessing the activity of the cardiac autonomic nervous system (ANS), making it particularly relevant for patients with atrial fibrillation (AF). Current guidelines recommend measuring the short-term HRV index through 5-minute electrocardiogram (ECG) recordings,^1^ which may pose challenges for widespread application. To address this limitation, ultra-short-term HRV (usHRV) has been introduced, allowing for HRV assessment from ECG recordings as brief as 10 to 30 seconds. The implementation of usHRV could significantly enhance large population-based studies and early risk stratification strategies, given that standard 10- to 30-second ECGs are performed by hundreds of millions of individuals each year.^2^ Moreover, wearable devices such as smartwatches can capture ECG data within this time frame, facilitating the measurement of usHRV.^3^ Despite the promising potential of these innovations to advance our understanding and prevention of cardiovascular diseases, there is currently a lack of research examining the relationship between usHRV and the risk of future cardiovascular events.^4^

Autonomy exhibits periodic fluctuations over time; however, the association between usHRV measured at different times of the day—particularly during day and night—and the risk of future cardiovascular events has not been thoroughly explored. While long-term HRV averages these fluctuations and provides insight into the overall state of the ANS, usHRV captures instantaneous changes in autonomic activity due to its brief sampling duration. This distinction underscores the need for further investigation into how usHRV variations throughout the day may influence cardiovascular risk.^5^ It may be more clinically meaningful to investigate the association between usHRV and AF at various times throughout the day. Understanding how usHRV fluctuates in relation to AF during different periods could provide valuable insights for risk assessment and management strategies.^6^ Furthermore, the regulatory function of the ANS tends to decline with age. UsHRV, a measure used to assess the ANS, shows notable variability across different age groups. Consequently, the association between usHRV and the risk of AF may vary among these age groups. Understanding these differences could enhance our ability to identify individuals at risk and tailor interventions accordingly.^7^

Therefore, the primary objective of this study was to investigate the patterns of usHRV and their association with AF risk at various times of the day. Additionally, the secondary aim was to examine the relationship between usHRV and AF risk across different age groups.

## Methods

### Study design and data sources

This retrospective cohort analysis included 48,416 participants drawn from the Medical Information Mart for Intensive Care IV (MIMIC-IV) database, with an observation period of 2 years. MIMIC-IV is a large, publicly available database that contains de-identified health-related data from patients admitted to the critical care units of Beth Israel Deaconess Medical Center, covering the years 2008 to 2019. The review committee at MIT and Beth Israel Deaconess Medical Center approved this database for research purposes, and a waiver for informed consent was granted.^8^ All procedures adhered to the ethical principles outlined in the Declaration of Helsinki.

### Study population

**Figure 1:**
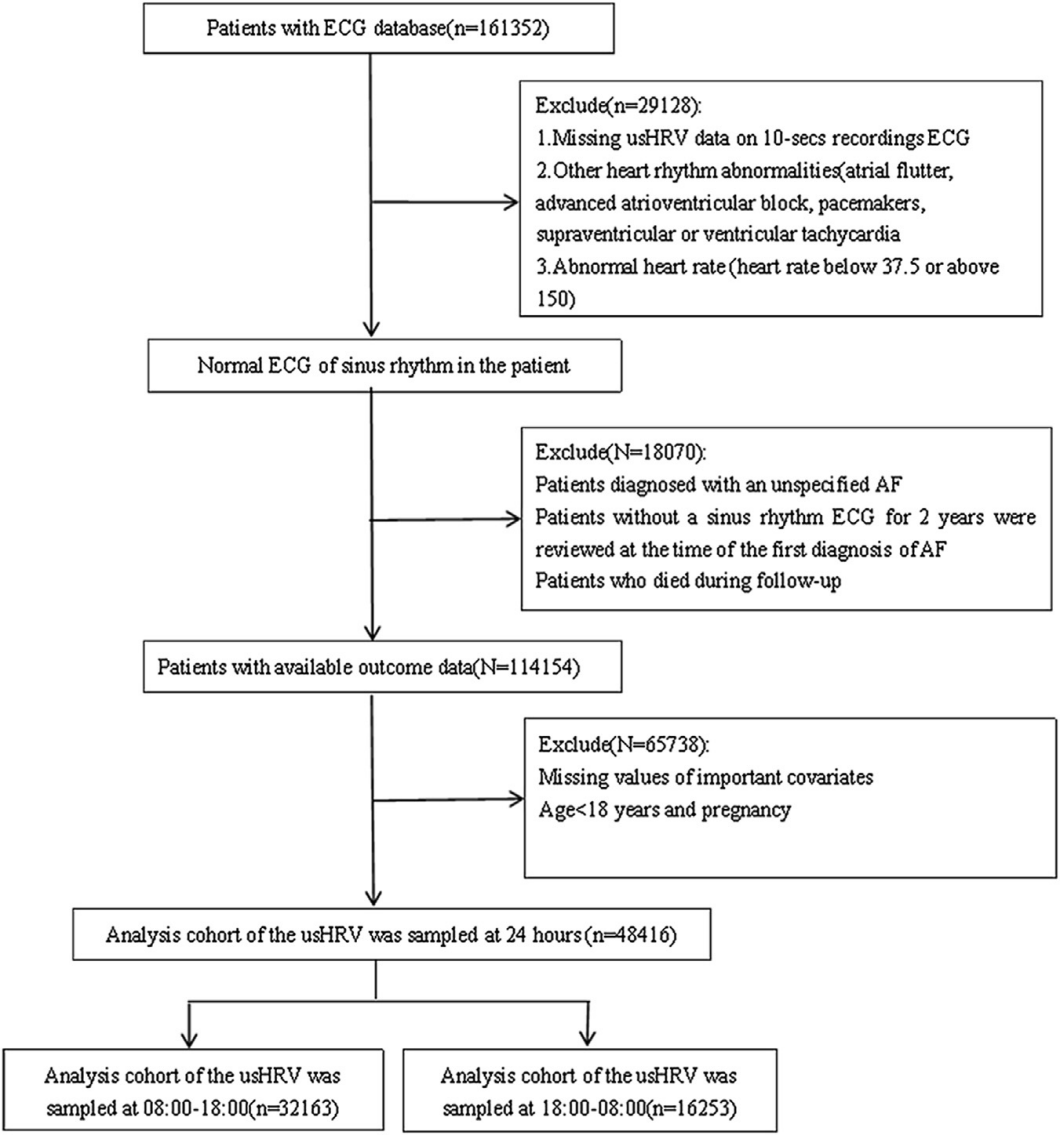
Flow of participants through the trial. AF = atrial fibrillation; ECG = electrocardiogram; usHRV = ultra-short-term heart rate variability.

We included a total of 161,352 patients who were admitted as outpatients, emergency patients, and inpatients from the MIMIC-IV and MIMIC-IV-ECG databases. Ultimately, 48,416 patients were selected for inclusion in this study. Figure 1 illustrates the flowchart detailing the exclusion criteria.^9^

### Assessment of usHRV and AF

#### ECG preprocessing

All ECGs were recorded from patients in the emergency department and inpatient settings using a bedside 12-lead ECG machine. Initially, we utilized the WFDB toolbox (v4.1.2) for Python (v3.11.8) available on the physical network to read the MIMIC-IV-ECG signals, obtaining 10-second ECG segments to calculate the RR intervals. We then compared these RR intervals against the official machine measurement table, filtering out any data with deviations >2% (ensuring 98% accuracy). Subsequently, we employed the hrv-analysis library (v1.0.3) in Python (Python Software Foundation, v3.11.8) to calculate time domain and frequency domain metrics in accordance with established guidelines.^10^

For noise filtering, raw ECG signals were preprocessed using a fourth-order Butterworth bandpass filter (0.5–40 Hz) to remove baseline wander and high-frequency noise. For RR interval extraction, RR intervals were detected using the Pan-Tompkins algorithm implemented in the WFDB toolbox. For quality control, RR intervals were validated against machine-measured values. Recordings with >2% deviation or >1 premature ventricular contraction (PVC)/premature atrial contraction (PAC). This library incorporates algorithms designed to identify and exclude abnormal beats, and PACs in 10-second segments were excluded.

#### HRV calculation

UsHRV time domain metrics were derived from the differences in RR intervals, including the standard deviation of RR intervals (SDNN), the standard deviation of successive differences (SDSD), and the root mean square of successive differences (RMSSD). We performed fast Fourier transformations to estimate the frequency metrics of usHRV, from which we computed the low frequency (LF), high frequency (HF), LF/HF ratio, LF normalized units (LFnu) (obtained by comparing LF with total power [TP]), HF normalized units (HFnu) (obtained by comparing HF with TP), TP, and very low frequency. All usHRV indices were log transformed to address skewed distributions, as outlined in previous studies.^11^, ^12^, ^13^

The time domain metrics were SDNN, SDSD, and RMSSD. The frequency domain metrics were LF (0.04–0.15 Hz), HF (0.15–0.40 Hz), and the LF/HF ratio. LFnu and HFnu were calculated as LFnu = LF/TP × 100.

HRV indices were computed using the hrv-analysis library (https://github.com/Aura-healthcare/hrv-analysis).

#### Ultra-short-term HRV

##### Detection of PVCs and PACs

The hrv-analysis library was utilized to detect and manage PVCs and PACs. This library incorporates algorithms designed to identify and exclude abnormal beats, such as PVCs and PACs, from the RR interval series. Specifically, it employs a threshold-based approach to detect beats that significantly deviate from the expected RR interval pattern.

##### Exclusion of abnormal beats

Once PVCs and PACs were detected, they were excluded from the RR interval series used to calculate usHRV metrics. The remaining normal RR intervals were used to compute the time domain (eg, SDNN, SDSD, RMSSD) and frequency domain (eg, LF, HF, LF/HF ratio) metrics. This approach ensured that the usHRV metrics reflected the underlying autonomic activity, rather than being skewed by ectopic beats.

##### Impact on analysis

Given the short duration of the ECG recordings (10 seconds), the presence of PVCs or PACs in a recording could significantly distort the usHRV metrics. Therefore, recordings with an HF of PVCs or PACs (eg, more than 1 abnormal beat in a 10-second recording) were excluded from the analysis to maintain the reliability of the results.

#### Atrial fibrillation

Incident AF was identified according to the International Classification of Diseases–Ninth Revision and International Classification of Diseases–Tenth Revision. If the first incident AF occurred prior to the baseline assessment, the participant was classified as having prevalent AF and was subsequently excluded from the analysis.^14^

### Covariates

SQL was utilized with PostgreSQL (version 16.2; PostgreSQL Global Development Group) to extract baseline characteristics, including age, sex, race, body mass index (BMI), hypertrophic cardiomyopathy (HCM), coronary artery disease (CAD), diabetes, heart failure, hypertension, and beta-blocker use.^4^^,^^15^

#### Statistical methods

UsHRV measurements were natural logarithmically transformed to normalize the distribution.^1^ Descriptive statistics of baseline characteristics were stratified according to incident AF status. Normally distributed continuous variables are presented as mean ± SD. Categorical variables are presented as frequency and percentage.

Cox proportional hazards models were used to estimate the relative hazards of AF per unit increase in log-transformed usHRV measures, with incremental adjustment for potential confounders. We compared nested Cox proportional hazards models using the Akaike information criterion and Bayesian information criterion to evaluate the incremental contribution of covariate adjustments.

Subgroup analysis was performed to examine the association between usHRV and AF risk based on subgroup variables. Interactions between subgroups were assessed using a likelihood ratio test.

A two-piece linear regression model with smoothing was used to analyze the threshold of association between age and usHRV after adjustment for variables. The likelihood ratio test and the bootstrap resampling method were used to identify inflection points. We also used stratified curve fitting to observe the association between usHRV and AF risk at different ages.

All statistical analyses were carried out using R Statistical Software (version 4.2.2; R Foundation for Statistical Computing) and Free Statistics Analysis Platform (version 1.9.2; http://www.clinicalscientists.cn/freestatistics).

## Results

### Baseline characteristics

At baseline, the mean age of the cohort was 56.7 ± 17.1 years, with 55.5% of participants identifying as women. During a mean follow-up period of 1.88 years, 7.5% (n = 3611 of 48,416) of patients developed AF. Those who developed AF were older and exhibited a higher prevalence of HCM, coronary artery disease, diabetes, heart failure, and hypertension (Table 1). To assess the association between usHRV and AF risk at different times, the sample was divided into 3 groups based on ECG recording times: 08:00 to 18:00, 18:00 to 08:00, and all-day recordings. In all 3 groups, participants who developed AF demonstrated lower usHRV frequency metrics compared with those without AF, with the exception of a higher HFnu observed in the AF group. However, there were no significant differences in the time domain metrics SDSD and RMSSD between the AF and non-AF cohorts, aside from a lower SDNN in the AF group (Table 1). The baseline characteristics for the 08:00 to 18:00 and 18:00 to 08:00 samples are presented in Supplemental Tables 1 and 2.

**Table 1:** Baseline characteristics of all-day samples

	Total (N = 48,416)	Incident atrial fibrillation		*P*
		No (n = 44,805)	Yes (n = 3611)	
Age, y	56.7 ± 17.1	55.5 ± 17.0	70.5 ± 12.4	<.001
Sex				<.001
Female	26,856 (55.5)	25,229 (56.3)	1627 (45.1)	
Male	21,560 (44.5)	19,576 (43.7)	1984 (54.9)	
Race/ethnicity				<.001
Caucasian	36,265 (74.9)	33,272 (74.3)	2993 (82.9)	
Asian	1888 (3.9)	1789 (4)	99 (2.7)	
African-American	7915 (16.3)	7517 (16.8)	398 (11)	
Other	2348 (4.8)	2227 (5)	121 (3.4)	
BMI, kg/m^2^	28.9 ± 7.3	28.9 ± 7.3	29.4 ± 7.6	<.001
HCM	124 (0.3)	100 (0.2)	24 (0.7)	<.001
CAD	3147 (6.5)	2724 (6.1)	423 (11.7)	<.001
Diabetes	5520 (11.4)	4981 (11.1)	539 (14.9)	<.001
Heart failure	2122 (4.4)	1647 (3.7)	475 (13.2)	<.001
Hypertension	22,547 (46.6)	20,557 (45.9)	1990 (55.1)	<.001
Βeta-blocker	3221 (6.7)	2618 (5.8)	603 (16.7)	<.001
Making ECG time				<.001
08:00 to 18:00	32,163 (66.4)	29,289 (65.4)	2874 (79.6)	
18:00 to 08:00	16,253 (33.6)	15,516 (34.6)	737 (20.4)	
RR interval, ms	814.1 ± 175.5	809.2 ± 174.2	876.0 ± 180.5	<.001
Log(SDNN)	1.1 ± 0.4	1.2 ± 0.4	1.1 ± 0.4	<.001
Log(SDSD)	1.1 ± 0.4	1.1 ± 0.4	1.1 ± 0.4	.397
Log(RMSSD)	1.1 ± 0.4	1.1 ± 0.4	1.1 ± 0.4	.382
Log(LF)	1.4 ± 0.9	1.4 ± 0.9	1.1 ± 0.9	<.001
Log(HF)	1.6 ± 0.8	1.6 ± 0.8	1.5 ± 0.8	<.001
Log(LF/HF)	–0.2 ± 0.5	–0.2 ± 0.5	–0.3 ± 0.6	<.001
Log(LFnu)	1.5 ± 0.4	1.5 ± 0.4	1.4 ± 0.4	<.001
Log(HFnu)	1.7 ± 0.2	1.7 ± 0.2	1.8 ± 0.2	<.001
Log(TP)	1.9 ± 0.8	1.9 ± 0.8	1.7 ± 0.8	<.001
Log(vLF)	0.5 ± 1.0	0.5 ± 1.0	0.2 ± 1.0	<.001

Values are mean ± SD or n (%).

BMI = body mass index; CAD = coronary artery disease; HCM = hypertrophic cardiomyopathy; HF = high frequency; HFnu = high-frequency normalized units; LF = low frequency; LFnu = low-frequency normalized units; RMSSD = root mean square of successive differences; SDNN = standard deviation of RR intervals; SDSD = standard deviation of successive differences; TP = total power; vLF = very low frequency.

### Association between usHRV of different times and AF risk

We employed a univariate Cox proportional hazards regression model to evaluate the association between each variable and the risk of AF (Supplemental Table 3). Additionally, we utilized a multivariable-adjusted Cox proportional hazards model to investigate the association between usHRV and AF risk during 3 different time periods: 08:00 to 18:00, 18:00 to 08:00, and all day (Tables 2, 3, and 4).

**Table 2:** Multivariate Cox proportional hazards model for usHRV, with relative hazards of AF with per-unit usHRV rise sampling from 8:00 to 18:00

	Model 1		Model 2		Model 3		Model 4	
	HR (95% CI)	*P* value	HR (95% CI)	*P* value	HR (95% CI)	*P* value	HR (95% CI)	*P* value
Log(SDNN)	0.9006 (0.811–0.999)	.049	0.897 (0.808–0.997)	.043	0.899 (0.810–0.997)	.044	0.91 (0.82–1.01)	.071
Log(SDSD)	1.18 (1.07–1.30)	.001	1.18 (1.07–1.30)	.001	1.16 (1.05–1.28)	.004	1.16 (1.05–1.28)	.004
Log(RMSSD)	1.18 (1.07–1.30)	.001	1.18 (1.06–1.30)	.002	1.16 (1.05–1.28)	.005	1.16 (1.05–1.28)	.005
Log(LF)	0.86 (0.82–0.89)	<.001	0.86 (0.82–0.89)	<.001	0.86 (0.83–0.90)	<.001	0.87 (0.84–0.91)	<.001
Log(HF)	0.94 (0.89–0.98)	.005	0.93 (0.89–0.98)	.005	0.94 (0.9–0.98)	.008	0.94 (0.90–0.99)	.017
Log(LF/HF)	0.76 (0.71–0.82)	<.001	0.76 (0.71–0.81)	<.001	0.77 (0.72–0.82)	<.001	0.79 (0.74–0.84)	<.001
Log(LFnu)	0.71 (0.65–0.77)	<.001	0.71 (0.65–0.77)	<.001	0.72 (0.66–0.78)	<.001	0.74 (0.67–0.80)	<.001
Log(HFnu)	2.43 (1.94–3.06)	<.001	2.44 (1.94–3.07)	<.001	2.36 (1.88–2.96)	<.001	2.26 (1.80–2.84)	<.001
Log(TP)	0.89 (0.85–0.94)	<.001	0.89 (0.85–0.93)	<.001	0.90 (0.86–0.94)	<.001	0.90 (0.86–0.95)	<.001
Log(vLF)	0.87 (0.84–0.91)	<.001	0.87 (0.84–0.91)	<.001	0.88 (0.84–0.91)	<.001	0.88 (0.85–0.92)	<.001

Model 1 was adjusted for age, sex, race, and BMI. Model 2 was adjusted for model 1 + HCM, CAD, and diabetes. Model 3 was adjusted for model 2 + heart failure and hypertension. Model 4: adjusted for model 3 + beta-blocker. HRs are expressed per-unit rise usHRV measures.

AF = atrial fibrillation; BMI = body mass index; CAD = coronary artery disease; CI = confidence interval; HCM = hypertrophic cardiomyopathy; HF = high frequency; HFnu = high-frequency normalized units; HR = hazard ratio; LF = low frequency; LFnu = low-frequency normalized units; RMSSD = root mean square of successive differences; SDNN = standard deviation of RR intervals; SDSD = standard deviation of successive differences; TP = total power; usHRV = ultra-short-term heart rate variability; vLF = very low frequency.

**Table 3:** Multivariate Cox proportional hazards model for usHRV, with relative hazards of AF with per-unit usHRV rise at all day

	Model 1		Model 2		Model 3		Model 4	
	HR (95% CI)	*P* value	HR (95% CI)	*P* value	HR (95% CI)	*P* value	HR (95% CI)	*P* value
Log(SDNN)	1.01 (0.92–1.1)	.906	1.01 (0.92–1.1)	.884	1.02 (0.93–1.11)	.713	1.03 (0.94–1.13)	.47
Log(SDSD)	1.3 (1.19–1.42)	<.001	1.3 (1.19–1.42)	<.001	1.29 (1.18–1.41)	<.001	1.3 (1.19–1.41)	<.001
Log(RMSSD)	1.3 (1.19–1.42)	<.001	1.3 (1.19–1.42)	<.001	1.29 (1.18–1.41)	<.001	1.3 (1.19–1.42)	<.001
Log(LF)	0.9 (0.86–0.93)	<.001	0.9 (0.86–0.93)	<.001	0.91 (0.88–0.94)	<.001	0.92 (0.89–0.95)	<.001
Log(HF)	0.98 (0.94–1.02)	.326	0.98 (0.94–1.02)	.338	0.99 (0.95–1.03)	.574	0.998 (0.96–1.04)	.875
Log(LF/HF)	0.77 (0.73–0.82)	<.001	0.78 (0.73–0.82)	<.001	0.79 (0.74–0.84)	<.001	0.8 (0.76–0.85)	<.001
Log(LFnu)	0.72 (0.67–0.78)	<.001	0.72 (0.67–0.78)	<.001	0.74 (0.68–0.8)	<.001	0.76 (0.7–0.82)	<.001
Log(HFnu)	2.28 (1.87–2.79)	<.001	2.27 (1.86–2.78)	<.001	2.18 (1.78–2.66)	<.001	2.08 (1.71–2.55)	<.001
Log(TP)	0.94 (0.9–0.98)	.003	0.94 (0.9–0.98)	.004	0.95 (0.91–0.99)	.017	0.96 (0.92–1.01)	.061
Log(vLF)	0.9 (0.87–0.93)	<.001	0.9 (0.87–0.93)	<.001	0.91 (0.88–0.94)	<.001	0.92 (0.89–0.95)	<.001

Model 1 was adjusted for age, sex, race, and BMI. Model 2 was adjusted for model 1 + HCM, CAD, and diabetes. Model 3 was adjusted for model 2 + heart failure and hypertension. Model 4 was adjusted for model 3 + beta-blocker. HRs are expressed per-unit rise usHRV measures.

AF = atrial fibrillation; BMI = body mass index; CAD = coronary artery disease; CI = confidence interval; HCM = hypertrophic cardiomyopathy; HF = high frequency; HFnu = high-frequency normalized units; HR = hazard ratio; LF = low frequency; LFnu = low-frequency normalized units; RMSSD = root mean square of successive differences; SDNN = standard deviation of RR intervals; SDSD = standard deviation of successive differences; TP = total power; usHRV = ultra-short-term heart rate variability; vLF = very low frequency.

**Table 4:** Multivariate Cox proportional hazards model for usHRV, with relative hazards of AF with per-unit usHRV rise sampling in 18:00 to 8:00

	Model 1		Model 2		Model 3		Model 4	
	HR (95% CI)	*P* value	HR (95% CI)	*P* value	HR (95% CI)	*P* value	HR (95% CI)	*P* value
Log(SDNN)	1.13 (0.92–1.37)	.243	1.14 (0.94–1.4)	.184	1.17 (0.96–1.43)	.111	1.22 (1.01–1.49)	.05
Log(SDSD)	1.46 (1.21–1.76)	<.001	1.47 (1.22–1.77)	<.001	1.47 (1.22–1.78)	<.001	1.50 (1.24–1.81)	<.001
Log(RMSSD)	1.45 (1.2–1.76)	<.001	1.46 (1.21–1.77)	<.001	1.47 (1.22–1.78)	<.001	1.50 (1.24–1.81)	<.001
Log(LF)	0.93 (0.86–1.01)	.084	0.94 (0.87–1.02)	.141	0.96 (0.89–1.04)	.359	0.98 (0.91–1.07)	.686
Log(HF)	1.01 (0.92–1.09)	.961	1.01 (0.92–1.1)	.835	1.03 (0.94–1.12)	.548	1.05 (0.96–1.14)	.316
Log(LF/HF)	0.82 (0.72–0.93)	.002	0.83 (0.73–0.94)	.005	0.85 (0.75–0.97)	.014	0.86 (0.76–0.98)	.028
Log(LFnu)	0.76 (0.64–0.9)	.002	0.77 (0.65–0.92)	.004	0.8 (0.68–0.96)	.013	0.82 (0.69–0.98)	.026
Log(HFnu)	1.74 (1.13–2.68)	.012	1.67 (1.08–2.57)	.021	1.59 (1.03–2.44)	.035	1.49 (0.97–2.29)	.067
Log(TP)	0.98 (0.89–1.07)	.599	0.99 (0.9–1.08)	.749	1.01 (0.92–1.1)	.895	1.03 (0.94–1.13)	.544
Log(vLF)	0.93 (0.86–1.01)	.055	0.94 (0.87–1.01)	.093	0.96 (0.89–1.03)	.278	0.98 (0.91–1.06)	.608

Model 1 was adjusted for age, sex, race, and BMI. Model 2 was adjusted for model 1 + HCM, CAD, and diabetes. Model 3 was adjusted for model 2 + heart failure and hypertension. Model 4 was adjusted for model 3 + beta-blocker. HRs are expressed per unit rise usHRV measures.

AF = atrial fibrillation; BMI = body mass index; CAD = coronary artery disease; CI = confidence interval; HCM = hypertrophic cardiomyopathy; HF = high frequency; HFnu = high-frequency normalized units; HR = hazard ratio; LF = low frequency; LFnu = low-frequency normalized units; RMSSD = root mean square of successive differences; SDNN = standard deviation of RR intervals; SDSD = standard deviation of successive differences; TP = total power; usHRV = ultra-short-term heart rate variability; vLF = very low frequency.

In accordance with previous literature, we included age, sex, race, BMI, HCM, CAD, diabetes, heart failure, hypertension, and beta-blocker use in our multivariable Cox proportional hazards model. In the fully adjusted Cox proportional hazards model for the period from 08:00 to 18:00 (model 4 in Table 2), we observed the following hazard ratios (HRs) for incident AF: 0.91 (95% confidence interval [CI] 0.82–1.01) for log(SDNN), 1.16 (95% CI 1.05–1.28) for log(SDSD), 1.16 (95% CI 1.05–1.28) for log(RMSSD), 0.87 (95% CI 0.84–0.91) for log(LF), 0.94 (95% CI 0.90–0.99) for log(HF), 0.79 (95% CI 0.74–0.84) for log(LF/HF), 0.74 (95% CI 0.67–0.80) for log(LFnu), 2.26 (95% CI 1.80–2.84) for log(HFnu), 0.90 (95% CI 0.86–0.95) for log(TP), and 0.88 (95% CI 0.85–0.92) for log(very low frequency). Interestingly, the time domain metrics of usHRV exhibited consistent associations with AF risk factors (Tables 2, 3, and 4). Conversely, the frequency domain metrics of usHRV appeared to serve as protective factors for AF stability, further reinforcing the need for a nuanced understanding of these variables (Tables 2, 3, and 4).

### Subgroup analysis and interaction

**Figure 2:**
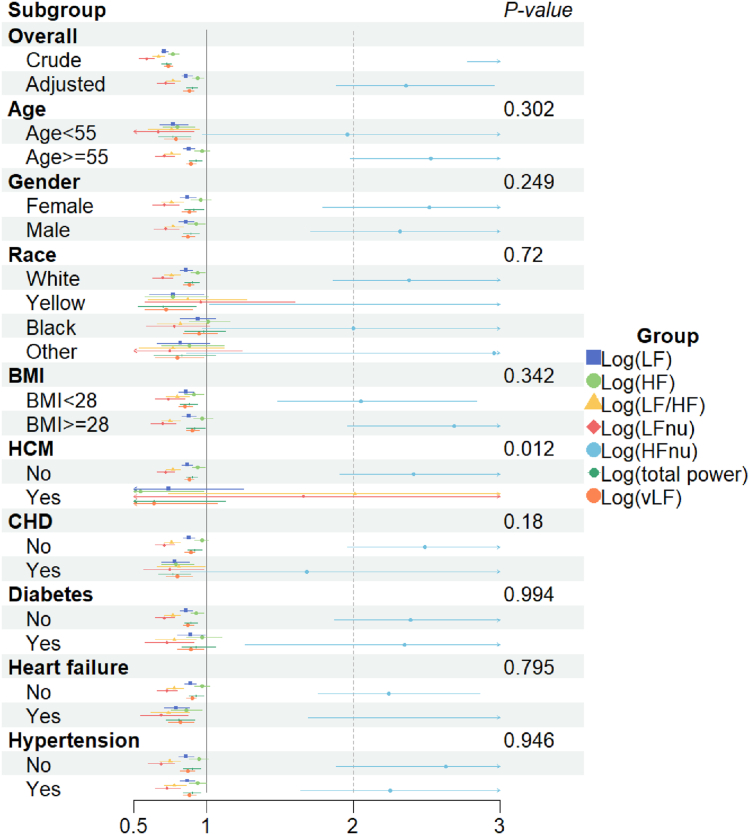
Subgroup analysis of ultra-short-term heart rate variability frequency domain. Adjustment factors included age, sex, race, body mass index, hypertrophic cardiomyopathy, coronary artery disease, diabetes, heart failure, and hypertension. HF = high frequency; HFnu = high-frequency normalized units; LF = low frequency; LFnu = low-frequency normalized units; vLF = very low frequency.

**Figure 3:**
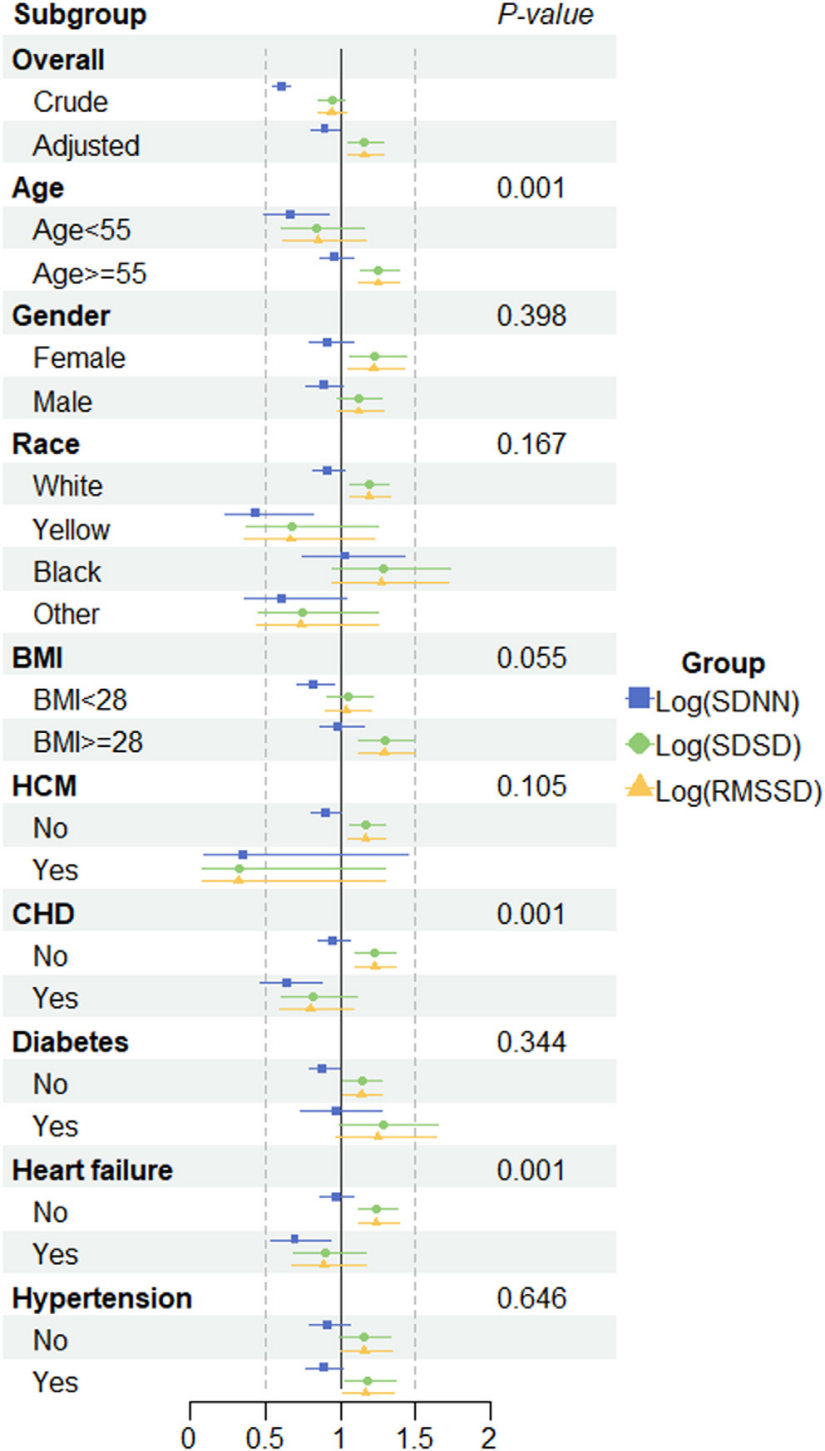
Subgroup analysis of ultra-short-term heart rate variability time domain. Adjustment factors included age, sex, race, body mass index, hypertrophic cardiomyopathy, coronary artery disease, diabetes, heart failure, and hypertension. RMSSD = root mean square of successive differences; SDNN = standard deviation of RR intervals; SDSD = standard deviation of successive differences.

**Figure 4:**
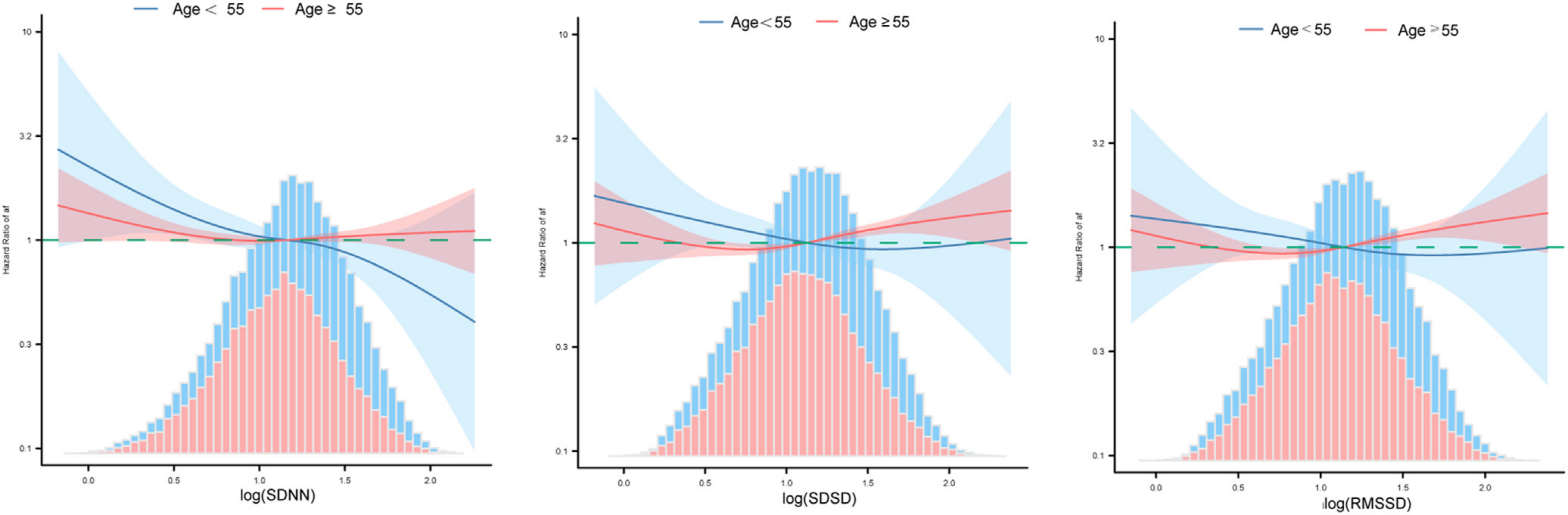
Association between ultra-short-term heart rate variability time domain metrics and incidence of atrial fibrillation at <55 and ≥55 years of age. Stratified curve-fitting of ultra-short-term heart rate variability time domain and incidence of atrial fibrillation was adjusted for age, sex, race, body mass index, hypertrophic cardiomyopathy, coronary artery disease, diabetes, heart failure, and hypertension. Dashed lines represent the hazard ratio of 1.00. RMSSD = root mean square of successive differences; SDNN = standard deviation of RR intervals; SDSD = standard deviation of successive differences.

Stratified and interaction analyses were performed using the 08:00 to 18:00 sample. In the frequency domain metrics of usHRV, consistent results were observed across stratifications by age, sex, race, BMI, HCM, CAD, diabetes, heart failure, and hypertension (Figure 2). In contrast, the time domain metrics of usHRV showed no significant differences in associations with sex, race, HCM, diabetes, or hypertension. Notably, we identified an interaction between usHRV and age in patients with AF (Figure 3). In individuals ≥55 years of age, both SDSD (HR 1.25, 95% CI, 1.13–1.39) and RMSSD (HR 1.25, 95% CI, 1.12–1.39) were identified as risk factors for AF. In contrast, among younger individuals, SDSD (HR 0.84, 95% CI, 0.61–1.15) and RMSSD (HR 0.85, 95% CI, 0.62–1.17) did not show statistically significant differences, potentially due to the limited sample size (only 281 cases of AF in individuals <55 years of age) (Figures 3 and 4). The age cutoff of 55 years was determined using linear inflection point analysis of age and usHRV time domain metrics (Table 5). Additionally, the associations of SDSD and RMSSD with the risk of AF exhibited interactive effects within the subgroups of CAD and heart failure (Figure 3). The reasons for these findings remain unclear and warrant further investigation.

**Table 5:** Threshold effect analysis of age on usHRV time domain (SDNN, SDSD, RMSSD)

Item	Break point (beta)	95% CI	*P* value
E_BK1	54.995	54.173 to 55.816	
slope1	–0.006	–0.006 to –0.005	<.001
slope2	–0.001	–0.002 to –0.001	<.001

Adjustment factors included age, sex, race, body mass index, hypertrophic cardiomyopathy, coronary artery disease, diabetes, heart failure, and hypertension.

CI = confidence interval; RMSSD = root mean square of successive differences; SDNN = standard deviation of RR intervals; SDSD = standard deviation of successive differences; usHRV = ultra-short-term heart rate variability.

### Sensitivity analysis

In our primary outcome analysis, we excluded patients who died directly. However, recognizing that death represents a competing risk for AF events, we conducted a sensitivity analysis by including deceased patients in the study population. We constructed a competing risk model and found that accounting for death as a competing risk event did not alter our main findings (Supplemental Table 4). In addition, we added a comparison of model performance metrics (Akaike information criterion, Bayesian information criterion) across sensitivity analyses in order to assess the effect of model (Supplemental Tables 6–14).

## Discussion

Previous studies have primarily focused on long-term HRV time domain metrics. In this large retrospective cohort study of MIMIC-IV patients, we were the first to explore the strength of the association between usHRV and the risk of AF across different time periods.^16^, ^17^, ^18^ Our findings indicate that frequency domain metrics of usHRV serve as protective factors against AF both during the day and at night (8:00 to 18:00, 18:00 to 8:00, and throughout the entire day), with LFnu, HFnu, and LF/HF demonstrating particularly strong performance.^7^^,^^19^, ^20^, ^21^ The following 3 frequency domain metrics warrant greater attention in future clinical practice: LFnu, HFnu, and LF/HF. These metrics exhibited robust protective effects in day, night, and all-day samples, while HFnu displayed significant hazardous effects. This suggests that these indices may be more sensitive to the state of the ANS, thereby offering greater clinical application value.^22^, ^23^, ^24^ Additionally, we discovered an interaction between usHRV time domain metrics and age with AF risk. This finding indicates that usHRV time domain metrics should be stratified by age, and the underlying mechanisms require further investigation.^25^, ^26^, ^27^

Among the frequency domain metrics of usHRV, HFnu has emerged as a robust and consistent risk factor for AF, highlighting the central role of vagal modulation in the pathogenesis of this arrhythmia. Experimental evidence from both animal models and human studies convincingly demonstrates the significant impact of the ANS on the initiation and perpetuation of AF.^28^^,^^29^ Specifically, vagal stimulation has been shown to induce and sustain AF, while the ablation of vagal inputs to the atria effectively abolishes this induction. Moreover, the success of interventions such as pulmonary vein isolation and the administration of class I antiarrhythmic drugs in reducing AF recurrence may be partially attributed to their ability to diminish vagal influence.^30^

While usHRV offers practical advantages for large-scale studies and wearable devices, its metrics are subject to significant limitations. First, usHRV metrics, particularly frequency domain measures (eg, LF, HF), are highly susceptible to transient noise, motion artifacts, and ectopic beats (eg, PVCs/PACs) in ultra-short recordings (10–30 seconds).^31^ For instance, a single ectopic beat in a 10-second ECG can drastically reduce RMSSD by up to 20%, leading to potential misinterpretation of autonomic tone. Second, unlike traditional short-term HRV measurements (which typically involve 5-minute recordings), there are currently no universally accepted standards for usHRV assessment. The variability in window length, artifact handling, and normalization methods complicates cross-study comparisons and hinders the establishment of standardized protocols.^32^ Last, the limited duration of recordings restricts the spectral resolution of LF components (0.04–0.15 Hz), which are critical for accurately assessing sympathetic activity. These factors underscore the need for cautious interpretation and further refinement of usHRV metrics in both clinical and research settings.^33^ A comparative analysis of usHRV and traditional HRV is now included as Supplemental Table 5.

The strength of our study lies in its relatively large population-based cohort, which provides sufficient statistical power to investigate the association between usHRV and AF.^34^^,^^35^ However, this study had several limitations that need to be considered. Despite efforts to control for many important confounders, the presence of unmeasured variables associated with AF risk cannot be completely excluded. Residual measured or unmeasured confounders may have influenced the results. Besides, We did not use a sliding window approach because the original ECG recordings in the MIMIC-IV database were only 10 seconds long, which is the standard duration for usHRV analysis. This may cause inaccurate usHRV measurements, but we used various ways of handling noise through the WFDB toolbox. Furthermore, We recognized that the separation of nocturnal and daytime data may introduce some limitations, particularly in terms of capturing the continuous dynamics of ANS activity over a 24-hour period. However, given the nature of the MIMIC-IV data (short-duration ECG recordings), this was the most feasible approach to explore diurnal variations in usHRV. Finally, Our study population was derived from the MIMIC-IV database, which predominantly includes critically ill patients admitted to intensive care units. Consequently, our findings may not generalize to healthier outpatient populations or individuals without critical illness. Future studies should validate these associations in non-ICU cohorts.

## Conclusion

The frequency domain metrics of usHRV exhibit strong stability, surpassing those derived from time domain metrics, and offer improved convenience compared with HRV. This makes them particularly notable for their clinical significance.

## Appendix Supplementary Data

STROBE checklist

Table E-value
