# Supplementary material for: Association between ultra-short-term heart rate variability of time fluctuation and atrial fibrillation: Evidence from MIMIC-IV
Source: Heart Rhythm O2. 2025 Mar 14;6(6):818–26. doi: 10.1016/j.hroo.2025.03.006 (PMC12287949; doi:10.1016/j.hroo.2025.03.006)
Supplement: Table E-value [file mmc2.docx]

**Table** E-value between usHRV indices and AF sampling in 8:00am-18:00pm.

| **Variable** | Item | point | lower | upper |
| --- | --- | --- | --- | --- |
| Log(SDSD) | RR | 1.16 | 1.05 | 1.28 |
|  | E-values | 1.59 | 1.28 | NA |
| Log(RMSSD) | RR | 1.16 | 1.05 | 1.28 |
|  | E-values | 1.59 | 1.28 | NA |
| Log(LF) | RR | 1.15 | 1.10 | 1.19 |
|  | E-values | 1.56 | 1.43 | NA |
| Log(HF) | RR | 1.06 | 1.01 | 1.11 |
|  | E-values | 1.32 | 1.11 | NA |
| Log(LF/HF) | RR | 1.27 | 1.19 | 1.35 |
|  | E-values | 1.85 | 1.67 | NA |
| Log(LFnu) | RR | 1.35 | 1.25 | 1.49 |
|  | E-values | 2.04 | 1.81 | NA |
| Log(HFnu) | RR | 2.26 | 1.8 | 2.84 |
|  | E-values | 3.95 | 3 | NA |
| Log(Total power) | RR | 1.11 | 1.05 | 1.16 |
|  | E-values | 1.46 | 1.29 | NA |
| Log(vLF) | RR | 1.14 | 1.09 | 1.18 |
|  | E-values | 1.53 | 1.39 | NA |
