# Supplementary material for: Association between ultra-short-term heart rate variability of time fluctuation and atrial fibrillation: Evidence from MIMIC-IV
Source: Heart Rhythm O2. 2025 Mar 14;6(6):818–26. doi: 10.1016/j.hroo.2025.03.006 (PMC12287949; doi:10.1016/j.hroo.2025.03.006)
Supplement: Supplementary Table 1 [file mmc3.docx]

| Variables | Total(n=32163) | Incident Atrial Fibrillation  No (n = 29289) Yes(n = 2874) | | *p* |
| --- | --- | --- | --- | --- |
| age | 57.6 ± 16.6 | 56.3 ± 16.4 | 70.3 ± 12.3 | < 0.001 |
| Gender, n(%) | | | | < 0.001 |
| Female | 18015 (56.0) | 16717 (57.1) | 1298 (45.2) |  |
| Male | 14148 (44.0) | 12572 (42.9) | 1576 (54.8) |  |
| race, n(%) | | | | < 0.001 |
| White | 24075 (74.9) | 21678 (74) | 2397 (83.4) |  |
| Yellow | 1262 (3.9) | 1176 (4) | 86 (3) |  |
| Black | 5270 (16.4) | 4980 (17) | 290 (10.1) |  |
| Other | 1556 (4.8) | 1455 (5) | 101 (3.5) |  |
| BMI, kg/m² | 29.1 ± 7.3 | 29.0 ± 7.3 | 29.4 ± 7.6 | < 0.001 |
| HCM, n(%) | 88 ( 0.3) | 70 (0.2) | 18 (0.6) | < 0.001 |
| CHD, n(%) | 1957 (6.1) | 1660 (5.7) | 297 (10.3) | < 0.001 |
| Diabetes, n(%) | 3562 (11.1) | 3172 (10.8) | 390 (13.6) | < 0.001 |
| Heart failure, n(%) | 1392 (4.3) | 1034 (3.5) | 358 (12.5) | < 0.001 |
| Hypertension, n(%) | 15267 (47.5) | 13759 (47) | 1508 (52.5) | < 0.001 |
| Β-bloker | 2142 (6.7) | 1713 (5.8) | 429 (14.9) | < 0.001 |
| Mean RR interval, ms | 829.4 ± 173.4 | 823.9 ± 171.8 | 886.3 ± 180.0 | < 0.001 |
| Log(SDNN) | 1.2 ± 0.4 | 1.2 ± 0.4 | 1.1 ± 0.4 | < 0.001 |
| Log(SDSD) | 1.1 ± 0.4 | 1.1 ± 0.4 | 1.1 ± 0.4 | 0.212 |
| Log(RMSSD) | 1.1 ± 0.4 | 1.1 ± 0.4 | 1.1 ± 0.4 | 0.220 |
| Log(LF) | 1.4 ± 0.9 | 1.4 ± 0.9 | 1.1 ± 0.9 | < 0.001 |
| Log(HF) | 1.7 ± 0.8 | 1.7 ± 0.8 | 1.5 ± 0.8 | < 0.001 |
| Log(LF/HF) | -0.2 ± 0.5 | -0.2 ± 0.5 | -0.3 ± 0.5 | < 0.001 |
| Log(LFnu) | 1.5 ± 0.4 | 1.5 ± 0.4 | 1.4 ± 0.4 | < 0.001 |
| Log(HFnu) | 1.7 ± 0.2 | 1.7 ± 0.2 | 1.8 ± 0.2 | < 0.001 |
| Log(Total power) | 1.9 ± 0.8 | 2.0 ± 0.8 | 1.7 ± 0.8 | < 0.001 |
| Log(vLF) | 0.5 ± 1.0 | 0.5 ± 1.0 | 0.2 ± 1.0 | < 0.001 |

**Table S1 Baseline characteristics of 08:00-18:00 samples**
