# Supplementary material for: Association between ultra-short-term heart rate variability of time fluctuation and atrial fibrillation: Evidence from MIMIC-IV
Source: Heart Rhythm O2. 2025 Mar 14;6(6):818–26. doi: 10.1016/j.hroo.2025.03.006 (PMC12287949; doi:10.1016/j.hroo.2025.03.006)
Supplement: Supplementary Table 2 [file mmc4.docx]

| Variables | Total(n=16253) | Incident Atrial Fibrillation  No (n = 15516) Yes(n = 737) | | *p* |
| --- | --- | --- | --- | --- |
| age | 54.9 ± 18.0 | 54.1 ± 17.9 | 71.3 ± 12.6 | < 0.001 |
| Gender, n(%) | | | | < 0.001 |
| Female | 8841 (54.4) | 8512 (54.9) | 329 (44.6) |  |
| Male | 7412 (45.6) | 7004 (45.1) | 408 (55.4) |  |
| race, n(%) | | | | < 0.001 |
| White | 12190 (75.0) | 11594 (74.7) | 596 (80.9) |  |
| Yellow | 626 (3.9) | 613 (4) | 13 (1.8) |  |
| Black | 2645 (16.3) | 2537 (16.4) | 108 (14.7) |  |
| Other | 792 (4.9) | 772 (5) | 20 (2.7) |  |
| BMI, kg/m² | 28.7 ± 7.4 | 28.7 ± 7.3 | 29.2 ± 7.7 | 0.058 |
| HCM, n(%) | 36 (0.2) | 30 (0.2) | 6 (0.8) | 0.005 |
| CHD, n(%) | 1190 (7.3) | 1064 (6.9) | 126 (17.1) | < 0.001 |
| Diabetes, n(%) | 1958 (12.0) | 1809 (11.7) | 149 (20.2) | < 0.001 |
| Heart failure, n(%) | 730 (4.5) | 613 (4) | 117 (15.9) | < 0.001 |
| Hypertension, n(%) | 7280 (44.8) | 6798 (43.8) | 482 (65.4) | < 0.001 |
| Β-bloker | 1079 (6.6) | 905 (5.8) | 174 (23.6) | < 0.001 |
| Mean RR interval, ms | 783.9 ± 175.7 | 781.4 ± 175.3 | 835.9 ± 176.9 | < 0.001 |
| Log(SDNN) | 1.1 ± 0.4 | 1.1 ± 0.4 | 1.1 ± 0.4 | < 0.001 |
| Log(SDSD) | 1.1 ± 0.4 | 1.1 ± 0.4 | 1.1 ± 0.4 | 0.128 |
| Log(RMSSD) | 1.1 ± 0.4 | 1.1 ± 0.4 | 1.1 ± 0.4 | 0.136 |
| Log(LF) | 1.3 ± 1.0 | 1.3 ± 1.0 | 1.0 ± 1.0 | < 0.001 |
| Log(HF) | 1.5 ± 0.9 | 1.5 ± 0.9 | 1.4 ± 0.9 | < 0.001 |
| Log(LF/HF) | -0.2 ± 0.5 | -0.2 ± 0.5 | -0.3 ± 0.6 | < 0.001 |
| Log(LFnu) | 1.5 ± 0.4 | 1.5 ± 0.4 | 1.4 ± 0.4 | < 0.001 |
| Log(HFnu) | 1.7 ± 0.2 | 1.7 ± 0.2 | 1.8 ± 0.2 | < 0.001 |
| Log(Total power) | 1.8 ± 0.9 | 1.8 ± 0.9 | 1.6 ± 0.8 | < 0.001 |
| Log(vLF) | 0.4 ± 1.0 | 0.4 ± 1.0 | 0.1 ± 1.0 | < 0.001 |

**Table S2 Baseline characteristics of 18:00-08:00 samples**
