# Supplementary material for: Association between ultra-short-term heart rate variability of time fluctuation and atrial fibrillation: Evidence from MIMIC-IV
Source: Heart Rhythm O2. 2025 Mar 14;6(6):818–26. doi: 10.1016/j.hroo.2025.03.006 (PMC12287949; doi:10.1016/j.hroo.2025.03.006)
Supplement: Supplementary Table 3 [file mmc5.docx]

| Variables | HR(95%CI) | *P* |
| --- | --- | --- |
| Age | 1.07 (1.06,1.07) | < 0.001 |
| Sex | 1.42 (1.19,1.69) | < 0.001 |
| Race, n (%) |  |  |
| White | 1 (Ref) |  |
| Yellow | 0.42 (0.21,0.8) | 0.009 |
| Black | 0.65 (0.5,0.86) | 0.002 |
| Other | 0.67 (0.42,1.07) | 0.096 |
| BMI | 1.02 (1.01,1.03) | < 0.001 |
| HCM | 5 (2.23,11.18) | < 0.001 |
| CHD | 3.26 (2.57,4.14) | < 0.001 |
| Diabetes,n (%) | 1.75 (1.39,2.21) | < 0.001 |
| Heart failure, n (%) | 4.45 (3.45,5.73) | < 0.001 |
| Hypertension, n (%) | 1.76 (1.47,2.11) | < 0.001 |
| Mean RRI | 1.0024 (1.0019,1.0029) | < 0.001 |
| Log (SDNN) | 0.63 (0.49,0.8) | < 0.001 |
| Log (SDSD) | 0.96 (0.76,1.21) | 0.704 |
| Log (RMSSD) | 0.97 (0.77,1.23) | 0.818 |
| Log (LF) | 0.72 (0.66,0.79) | < 0.001 |
| Log (HF) | 0.77 (0.69,0.86) | < 0.001 |
| Log (LF/HF) | 0.7 (0.59,0.82) | < 0.001 |
| Log (LFnu) | 0.62 (0.5,0.77) | < 0.001 |
| Log (HFnu) | 3.01 (1.73,5.24) | < 0.001 |
| Log (Total power) | 0.73 (0.65,0.81) | < 0.001 |
| Log (vLF) | 0.74 (0.68,0.81) | < 0.001 |

**Table S3.** Univariate COX proportional hazards regression model about the incidence of AF.
