# Supplementary material for: Association between ultra-short-term heart rate variability of time fluctuation and atrial fibrillation: Evidence from MIMIC-IV
Source: Heart Rhythm O2. 2025 Mar 14;6(6):818–26. doi: 10.1016/j.hroo.2025.03.006 (PMC12287949; doi:10.1016/j.hroo.2025.03.006)
Supplement: Supplementary Table 4 [file mmc6.docx]

|  | **Model 1** | | **Model 2** | | **Model 3** | |
| --- | --- | --- | --- | --- | --- | --- |
|  | **HR (95% CI)** | **P-value** | **HR (95% CI)** | **P-value** | **HR (95% CI)** | **P-value** |
| Log(SDNN) | 1.01 (0.91 - 1.12) | 0.833 | 1.01 (0.91 - 1.12) | 0.914 | 1.02 (0.92 - 1.13) | 0.716 |
| Log(SDSD) | 1.28 (1.16 - 1.41) | <0.001 | 1.25 (1.13 - 1.38) | <0.001 | 1.26 (1.14 - 1.39) | <0.001 |
| Log(RMSSD) | 1.28 (1.16 - 1.42) | <0.001 | 1.26 (1.14 - 1.39) | <0.001 | 1.26 (1.14 - 1.39) | <0.001 |
| Log(LF) | 0.90 (0.87 - 0.94) | <0.001 | 0.91 (0.87 - 0.95) | <0.001 | 0.92 (0.88 - 0.96) | <0.001 |
| Log(HF) | 0.98 (0.94 - 1.03) | 0.527 | 0.99 (0.94 - 1.03) | 0.537 | 0.99 (0.95 - 1.04) | 0.73 |
| Log(LF/HF) | 0.78 (0.74 - 0.84) | <0.001 | 0.80 (0.75 - 0.85) | <0.001 | 0.81 (0.76 - 0.86) | <0.001 |
| Log(LFnu) | 0.74 (0.68 - 0.80) | <0.001 | 0.75 (0.69 - 0.82) | <0.001 | 0.77 (0.70 - 0.84) | <0.001 |
| Log(HFnu) | 2.28 (1.80 - 2.88) | <0.001 | 2.17 (1.72 - 2.75) | <0.001 | 2.11 (1.67 - 2.67) | <0.001 |
| Log(Total power) | 0.94 (0.90 - 0.99) | 0.02 | 0.95 (0.90 - 0.99) | 0.027 | 0.96 (0.91 - 0.99) | 0.059 |
| Log(vLF) | 0.91 (0.88 - 0.95) | <0.001 | 0.91 (0.88 - 0.95) | <0.001 | 0.92 (0.88 - 0.96) | <0.001 |

**Table S4.** Multivariate cox proportional hazards model considering competing risks (all-cause mortality) for usHRV, relative hazards of AF with Per unit usHRV rise sampling in 8:00am-18:00pm.

Model 1: adjusted for age, sex, race, and BMI.

Model 2: adjusted for model 1 + HCM, CHD, diabetes, heart failure, hypertension.

Model 3: adjusted for model 2 + beta-blocker.

Hazard ratios (HR) are expressed per unit rise usHRV measures. AF: atrial fibrillation; CI: confidence interval; other abbreviations as in Table 1.
