# Supplementary material for: Association between ultra-short-term heart rate variability of time fluctuation and atrial fibrillation: Evidence from MIMIC-IV
Source: Heart Rhythm O2. 2025 Mar 14;6(6):818–26. doi: 10.1016/j.hroo.2025.03.006 (PMC12287949; doi:10.1016/j.hroo.2025.03.006)
Supplement: Supplementary Table 5 [file mmc7.docx]

### Table S5 **Strengths and Weaknesses of usHRV vs. Traditional HRV**

| **Aspect** | **usHRV** | **Traditional HRV** |
| --- | --- | --- |
| Recording Duration | 10–30 seconds | 5–24 hours |
| Noise Sensitivity | High (single artifact distorts metrics) | Moderate (artifacts averaged out) |
| Spectral Resolution | Limited (poor LF reliability) | High (resolves LF/HF accurately) |
| Clinical Utility | Rapid screening, wearables | Gold standard for ANS assessment |
| Standardization | No consensus | Established guidelines (ESC/NASPE) |
