# Supplementary material for: Association between ultra-short-term heart rate variability of time fluctuation and atrial fibrillation: Evidence from MIMIC-IV
Source: Heart Rhythm O2. 2025 Mar 14;6(6):818–26. doi: 10.1016/j.hroo.2025.03.006 (PMC12287949; doi:10.1016/j.hroo.2025.03.006)
Supplement: Supplementary Table 6-15 [file mmc8.docx]

| **Model** | **Adjustments** | **AIC** | **BIC** |
| --- | --- | --- | --- |
| Model 1 | age, sex, race, and BMI | 57829.24 | 57888.50 |
| Model 2 | model 1 + HCM, CHD, diabetes | 57828.91 | 57913.58 |
| Model 3 | model 2 + heart failure, hypertension | 57647.85 | 57749.45 |
| Model 4 | model 3 + beta-blocker | 57534.18 | 57567.32 |

**Table S6** Model fit statistics for log(SDNN)

**Table S7** Model fit statistics for log(SDSD)

**Table S8** Model fit statistics for log(RMSSD)

**Table S9** Model fit statistics for log(LF)

| **Model** | **Adjustments** | **AIC** | **BIC** |
| --- | --- | --- | --- |
| Model 1 | age, sex, race, and BMI | 57804.86 | 57864.13 |
| Model 2 | model 1 + HCM, CHD, diabetes | 57805.60 | 57890.27 |
| Model 3 | model 2 + heart failure, hypertension | 57647.47 | 57749.06 |
| Model 4 | model 3 + beta-blocker | 57539.58 | 57649.64 |

**Table S10** Model fit statistics for log(HF)

| **Model** | **Adjustments** | **AIC** | **BIC** |
| --- | --- | --- | --- |
| Model 1 | age, sex, race, and BMI | 57828.87 | 57888.13 |
| Model 2 | model 1 + HCM, CHD, diabetes | 57828.32 | 57912.98 |
| Model 3 | model 2 + heart failure, hypertension | 57627.72 | 57729.31 |
| Model 4 | model 3 + beta-blocker | 57556.43 | 57666.49 |

| **Model** | **Adjustments** | **AIC** | **BIC** |
| --- | --- | --- | --- |
| Model 1 | age, sex, race, and BMI | 57805.67 | 57864.93 |
| Model 2 | model 1 + HCM, CHD, diabetes | 57803.92 | 57888.58 |
| Model 3 | model 2 + heart failure, hypertension | 57627.15 | 57728.74 |
| Model 4 | model 3 + beta-blocker | 57556.44 | 57666.50 |

| **Model** | **Adjustments** | **AIC** | **BIC** |
| --- | --- | --- | --- |
| Model 1 | age, sex, race, and BMI | 57804.79 | 57864.05 |
| Model 2 | model 1 + HCM, CHD, diabetes | 57805.52 | 57805.52 |
| Model 3 | model 2 + heart failure, hypertension | 57627.48 | 57729.08 |
| Model 4 | model 3 + beta-blocker | 57535.52 | 57645.58 |

**Table S11** Model fit statistics for log(LF/HF)

**Table S12** Model fit statistics for log(LFnu)

**Table S13** Model fit statistics for log(HFnu)

| **Model** | **Adjustments** | **AIC** | **BIC** |
| --- | --- | --- | --- |
| Model 1 | age, sex, race, and BMI | 57785.32 | 57844.59 |
| Model 2 | model 1 + HCM, CHD, diabetes | 57784.76 | 57869.42 |
| Model 3 | model 2 + heart failure, hypertension | 57601.56 | 57703.16 |
| Model 4 | model 3 + beta-blocker | 57513.45 | 57623.51 |

**Table S14** Model fit statistics for log(Total power)

| **Model** | **Adjustments** | **AIC** | **BIC** |
| --- | --- | --- | --- |
| Model 1 | age, sex, race, and BMI | 57806.11 | 57865.38 |
| Model 2 | model 1 + HCM, CHD, diabetes | 57804.31 | 57888.98 |
| Model 3 | model 2 + heart failure, hypertension | 57625.97 | 57727.57 |
| Model 4 | model 3 + beta-blocker | 57552.91 | 57662.97 |

| **Model** | **Adjustments** | **AIC** | **BIC** |
| --- | --- | --- | --- |
| Model 1 | age, sex, race, and BMI | 57777.09 | 57836.35 |
| Model 2 | model 1 + HCM, CHD, diabetes | 57776.49 | 57861.15 |
| Model 3 | model 2 + heart failure, hypertension | 57642.80 | 57744.39 |
| Model 4 | model 3 + beta-blocker | 57538.05 | 57648.11 |

| **Model** | **Adjustments** | **AIC** | **BIC** |
| --- | --- | --- | --- |
| Model 1 | age, sex, race, and BMI | 57779.05 | 57838.31 |
| Model 2 | model 1 + HCM, CHD, diabetes | 57778.45 | 57863.11 |
| Model 3 | model 2 + heart failure, hypertension | 57604.52 | 57706.12 |
| Model 4 | model 3 + beta-blocker | 57517.20 | 57627.26 |

**Table S15** Model fit statistics for log(vLF)

| **Model** | **Adjustments** | **AIC** | **BIC** |
| --- | --- | --- | --- |
| Model 1 | age, sex, race, and BMI | 57823.72 | 57882.98 |
| Model 2 | model 1 + HCM, CHD, diabetes | 57822.66 | 57907.32 |
| Model 3 | model 2 + heart failure, hypertension | 57610.42 | 57712.01 |
| Model 4 | model 3 + beta-blocker | 57522.93 | 57632.99 |
